# The Removal of Endo- and Enterotoxins From Bacteriophage Preparations

**DOI:** 10.3389/fmicb.2019.01674

**Published:** 2019-07-23

**Authors:** Ville Hietala, Jenni Horsma-Heikkinen, Annelie Carron, Mikael Skurnik, Saija Kiljunen

**Affiliations:** ^1^Department of Bacteriology and Immunology, Human Microbiome Research Program, Faculty of Medicine, University of Helsinki, Helsinki, Finland; ^2^Division of Clinical Microbiology, HUSLAB, Helsinki University Hospital, Helsinki, Finland

**Keywords:** antibiotic resistance, bacteriophage, phage therapy, endotoxin, enterotoxin

## Abstract

The production of phages for therapeutic purposes demands fast, efficient and scalable purification procedures. Phage lysates have a wide range of impurities, of which endotoxins of gram-negative bacteria and protein toxins produced by many pathogenic bacterial species are harmful to humans. The highest allowed endotoxin concentration for parenterally applied medicines is 5 EU/kg/h. The aim of this study was to evaluate the feasibility of different purification methods in endotoxin and protein toxin removal in the production of phage preparations for clinical use. In the purification assays, we utilized three phages: *Escherichia* phage vB_EcoM_fHoEco02, *Acinetobacter* phage vB_ApiM_fHyAci03, and *Staphylococcus* phage vB_SauM_fRuSau02. The purification methods tested in the study were precipitation with polyethylene glycol, ultracentrifugation, ultrafiltration, anion exchange chromatography, octanol extraction, two different endotoxin removal columns, and different combinations thereof. The efficiency of the applied purification protocols was evaluated by measuring phage titer and either endotoxins or staphylococcal enterotoxins A and C (SEA and SEC, respectively) from samples taken from different purification steps. The most efficient procedure in endotoxin removal was the combination of ultrafiltration and EndoTrap HD affinity column, which was able to reduce the endotoxin-to-phage ratio of vB_EcoM_fHoEco02 lysate from 3.5 × 10^4^ Endotoxin Units (EU)/10^9^ plaque forming units (PFU) to 0.09 EU/10^9^ PFU. The combination of ultrafiltration and anion exchange chromatography resulted in ratio 96 EU/10^9^ PFU, and the addition of octanol extraction step into this procedure still reduced this ratio threefold. The other methods tested either resulted to less efficient endotoxin removal or required the use of harmful chemicals that should be avoided when producing phage preparations for medical use. Ultrafiltration with 100,000 MWCO efficiently removed enterotoxins from vB_SauM_fRuSau02 lysate (from 1.3 to 0.06 ng SEA/10^9^ PFU), and anion exchange chromatography reduced the enterotoxin concentration below 0.25 ng/ml, the detection limit of the assay.

## Introduction

The antibiotic resistance of pathogenic bacteria is recognized as one of the major global health threats of our time (World Health Organization [WHO], 2018). Some experts even state that we are already entering a post-antibiotic era, where medical treatments such as operations need to be reconsidered due to high risk of infection (Fowler et al., 2014). Phage therapy, i.e., the treatment of bacterial infections with bacteriophages, is one of the possible alternative therapies that can be used to cure patients for whom antibiotics are not an option (Kutter et al., 2010, 2015; Abedon et al., 2011).

Phages themselves are considered safe for humans (Speck and Smithyman, 2016). However, phage lysates may contain many kinds of harmful by-products, especially endotoxins of gram-negative bacteria and protein toxins produced by many pathogenic bacterial species. Endotoxins are composed of Lipid A, the hydrophobic anchor of lipopolysaccharides (LPS) forming the outmost layer of gram-negative bacteria. Endotoxins are highly immunogenic and if present in large quantities, they may via cytokine signaling cause septic (endotoxic) shock leading to intravascular coagulation, multiple organ failure, and even death (Raetz and Whitfield, 2002). The endotoxin levels in medicinal products are strictly regulated, the highest permitted amount being 5.0 Endotoxin Units (EU)/kg/h for intravenous and 0.2 EU/kg/h for intrathecal products (Council of Europe, 2005).

Many pathogenic bacteria produce protein toxins that they excrete into their environment. These toxins are often important virulence factors and they have a wide variety of effects in humans, depending on the bacterial species and the type of infection. Examples of bacterial toxins include Shiga toxin of *Escherichia coli* (Plunkett et al., 1999; Chou et al., 2013), botulinum toxin of *Clostridium botulinum* (Poulain and Popoff, 2019), and enterotoxins of *Staphylococcus aureus* (Betley and Mekalanos, 1985; Dinges et al., 2000). Bacterial toxins are often extremely potent, amounts as small as tens to hundreds of nanograms can cause disease (Asao et al., 2003; Pirazzini et al., 2017).

The traditional way to purify phages is PEG precipitation followed by several rounds of chloroform extractions, ultracentrifugation with cesium chloride (CsCl) gradient, and finally dialysis to remove CsCl. For therapeutic purposes, this is a problematic approach for several reasons: First, the amount of chloroform is strictly regulated in medical products and, according to ICH harmonized guideline Q3C(R6), the permissible daily exposure should not exceed 0.6 mg/day. Therefore, the use of chloroform would necessitate the analysis of residual solvent concentration. Second, also CsCl is considered safety risk (Food and Drug Administration., 2018), even though the residual CsCl concentration in phage products would probably be irrelevant (Guerin et al., 2015). Third, this process is very laborious and slow.

More recently, several alternative purification methods have been applied. Szermer-Olearnik and Boratynski (2015) showed that extraction with 1-octanol efficiently removed endotoxins from phage lysates (Szermer-Olearnik and Boratynski, 2015). Later, Bonilla et al. (2016) developed the method further by introducing a vacuum-based method to remove the residual solvent. The phage recoveries in these two studies were typically ∼50%. Extraction with the detergent deoxycholate (DOC) was shown to remove LPS from phage preparation without significant reduction in phage titers (Hashemi et al., 2013), but the harmfulness of DOC to humans may limit its use for therapeutic purposes. A number of studies have shown that different chromatographic methods can be used to purify phages. Anion exchange chromatography with monolithic columns having weak (DEAE) or strong (QA) anion exchange resins is a widely applied chromatographic method, with usually fairly good phage recovery (Smrekar et al., 2008, 2011; Adriaenssens et al., 2012). However, the emphasis in these studies done with anion exchange chromatography has mostly been in the phage yield, and the endotoxin removal has not been examined. Boratynski et al. (2004) showed that a protocol containing ultrafiltration, gel filtration and cellufine sulfate -based affinity chromatography produced a phage preparation with very low endotoxin concentration, but the phage yield in the process was less than 3% (Boratynski et al., 2004). Commercial endotoxin removal columns have also been used to purify phages, however, the results have been somewhat confusing. Merabishvili et al. (2009) used EndoTrap Blue column to remove endotoxins from a therapeutic phage cocktail, but as the authors found the Limulus amoebocyte lysate (LAL) endotoxin analysis kit inappropriate for phage products, the outcome was only measured with pyrogenicity assay using rabbits (Merabishvili et al., 2009). In another study by Cooper et al. (2014), the purification of a *Pseudomonas aeruginosa* phage cocktail with the EndoTrap Blue column resulted only in a minimal reduction of the endotoxin levels (Cooper et al., 2014). As shown in the aforementioned examples, phage purification studies done so far have mostly focused on phage yield and endotoxin removal. To our knowledge, there are no studies showing how bacterial toxins are removed from phage preparations during the phage purification process.

The aim of this work was to study the applicability of the different phage purification methods in the preparation of clinical phage products. We wanted to establish a purification process that would efficiently remove endotoxins and bacterial protein toxins while retaining high phage yield. In addition to the phage recovery and product purity, our criteria for the process optimization were the laboriousness of the process and the possibility for scaling-up. The methods tested for purification were precipitation with polyethylene glycol, ultracentrifugation, ultrafiltration, anion exchange chromatography, octanol extraction, two different endotoxin removal columns, and different combinations thereof. The endotoxin removal experiments were mostly performed with an *Escherichia* phage vB_EcoM_fHoEco02, a member of *Tequatrovirus* genus (Kiljunen et al., 2018). To study whether the endotoxin affinity columns can also remove endotoxins of other gram-negative bacteria than *E. coli*, confirmatory tests were done with vB_ApiM_fHyAci03 infecting *Acinetobacter pittii* (Pulkkinen et al., 2019). Since LAL endotoxin measurement kit is sensitive to impurities often present in phage products, we used EndoLISA assay (Hyglos) which is supposed to be less error-prone. To analyze the removal of protein toxins from phage preparation, we utilized staphylococcal phage vB_SauM_fRuSau02 grown in a *S. aureus* strain known to produce staphylococcal enterotoxins (Leskinen et al., 2017).

## Materials and Methods

### Bacterial Strains, Phages and Media

Three clinical bacterial strains obtained from The Hospital District of Helsinki and Uusimaa Laboratories (HUSLAB), Finland, were used in this work: *E. coli* 123738 (Kiljunen et al., 2018), *A. pittii* #5565 (Pulkkinen et al., 2019) and *S. aureus* 13KP (Leskinen et al., 2017). The phages cultured in these strains were vB_EcoM_fHoEco02 (fHoEco02) (Kiljunen et al., 2018), vB_ApiM_fHyAci03 (fHyAci03) (Pulkkinen et al., 2019), and vB_SauM_fRuSau02 (fRuSau02) (Leskinen et al., 2017), respectively.

All bacterial and phage incubations were carried out at 37°C using Luria Broth (LB) medium (Sambrook and Russell, 2001). Soft agar medium included additionally 0.4% (w/v) agar (Becton Dickinson), and LB agar plates were solidified with 1.5% (w/v) of agar. fHoEco02 and fHyAci03 lysates were produced by liquid culture method and fRuSau02 lysate from semiconfluent plates otherwise as described elsewhere (Sambrook and Russell, 2001) but without using chloroform. All phage lysates were filtered through 0.22 μm PES Rapid Flow Bottle Top filter (Nalgene). Unless otherwise stated, SM buffer (100 mM NaCl, 10 mM MgSO_4_, 50 mM Tris–HCl, pH 7.5, and 0.01% (w/v) gelatin) (Sambrook and Russell, 2001) was used as phage buffer.

### Phage Purification

#### PEG Precipitation, Ultrafiltration, and Ultracentrifugation

Phage precipitation with PEG 8000 and subsequent chloroform extractions were done as described elsewhere (Sambrook and Russell, 2001). Briefly: 30 ml of fHoEco02 lysate was treated with NaCl (1M), RNAseA (1.2 μg/ml) DNAseI (1 μg/ml) for 1 h on ice, after which the solution was centrifuged with Avanti J-26 XPI centrifuge (Beckman Coulter) at 8,739 × *g* and +4°C for 20 min. 8 g of PEG was dissolved in the supernatant and the solution was incubated o/n at +4°C. The solution was centrifuged as above and the pellet was dissolved into 5 ml of TM buffer (10 mM MgSO_4_, 50 mM Tris, and pH 7.5). The solution was extracted with 1 VOL chloroform for four times, and the centrifugations were done with Sl-16R centrifuge (Thermo scientific) at 3500 × *g* and RT for 17 min.

For ultrafiltration, Vivaspin ultrafiltration columns with 100,000 MWCO polyethersulfone (PES) membrane (Sartorius) were used. The phage samples were concentrated by centrifugation to one tenth of the initial volume and washed by restoring the original volume with a desired buffer and repeating the centrifugation step. Finally, the original volume was restored by adding the same buffer as was used for washing. The samples were centrifuged at 3500 × *g* at RT using the Sl-16R centrifuge (Thermo scientific).

For ultracentrifugation through sucrose density gradient, 2 ml of PEG-precipitated or ultrafiltrated phage sample was layered on top of 3 ml of pre-formed 5–20% sucrose gradient. The samples were centrifuged at 194,432 × *g* and +4°C for 2 h with Optima L-80 XP ultracentrifuge and SW55Ti rotor (Beckman Coulter), after which the phage pellet was resuspended into 500 μl of SM-buffer.

#### Extraction With 1-Octanol

Extraction with 1-octanol was basically carried out as described earlier (Szermer-Olearnik and Boratynski, 2015). 7 ml of phage lysate was treated with 5 ml of 99% 1-octanol (Acros organics) and the phases were separated by centrifuging at 4,000 × *g* and +4°C for 10 min. The removal of octanol was enhanced by repeating the centrifugation step two additional times.

#### Anion Exchange Chromatography

Prior to anion exchange chromatography (AEX), 5 ml of phage lysate or 4 ml of octanol-treated phage lysate was ultrafiltrated to 1 volume of chromatography buffer (20 mM Tris–HCl, pH 7.5) as above and filtered with 0.2 μm Minisart RC Syringe Filter (Sartorius). For AEX, CIM QA-1 tube monolithic column with 6-μm pore size (BIA Separations) and Äkta Purifier (GE Healthcare) were used. The run conditions for fHoEco02 were optimized by running a linear NaCl gradient in the chromatography buffer and determining the NaCl concentration that was needed to elute the phage; the conditions for fRuSau02 runs were determined earlier (Leskinen et al., 2017). To purify fHoEco02, 900 μl of ultrafiltrated phage solution was injected into the column. In purification schemes where the AEX purified phages were further purified with endotoxin affinity columns, 1.8 and 3.0 ml of ultrafiltrated fHoEco02 solutions were applied to AEX prior to EndoTrap HD and Pierce columns, respectively. A step gradient with chromatography buffer having 260 and 350 mM NaCl was used to wash and elute the phage, respectively. For fRuSau02, a 500-μl sample was injected into the column. 350 mM NaCl was used for washing and 550 mM NaCl for elution. 1 ml fractions were collected throughout the runs. The phage fractions (3–4 ml in total) were pooled, a 100 to 150-μl sample was withdrawn for analysis, and the remaining phage sample was ultrafiltrated into SM buffer to a volume corresponding to the volume that was injected in the column.

#### Endotoxin Affinity Columns

Two commercial endotoxin affinity columns were used for endotoxin removal: EndoTrap HD 1 ml column (LIONEX) and Pierce high-capacity endotoxin removal spin 1 ml column (Thermo Scientific). For EndoTrap HD, 750 μl of ultrafiltrated or AEX purified phage in SM buffer supplemented with 0.1 mM CaCl_2_ was run through the column using gravity flow. The void volume of 300 μl was taken in a separate tube, after which the phage sample of ∼1.3 ml was collected. For Pierce column, 5 ml of ultrafiltrated phage lysate in SM buffer supplemented with 400 mM, NaCl, or 2 ml of AEX -purified and ultrafiltrated phage in the same buffer was applied in the column and incubated at +4°C for 1 h. Approximately 4.8 ml of the phage solution was collected by centrifugation at 500 × *g* at RT for 1 min.

### The Analysis of Phage Samples

Every purification scheme in this study was repeated three individual times. The phage titer and endotoxin concentration of samples taken from every step were determined, and mean and standard deviation (SD) of the results were calculated. Phage titers were determined with the standard double-layer assay as described elsewhere (Sambrook and Russell, 2001). Endotoxin concentrations were determined with EndoLISA assay (Hyglos) and staphylococcal enterotoxins were measured with RIDASCREEN^®^ SET A–E kit (r-biopharm) using staphylococcal enterotoxin A (Merck) and C2 (Abcam) as standards. For both assays, fluorescence values were measured using HIDEX sense microplate reader with 0.5.35.0 platereader software (Hidex).

## Results

### Removal of *E. coli* Endotoxins by PEG Precipitation and Sucrose Gradient Ultracentrifugation

The traditional procedure for phage purification is the combination of PEG precipitation, chloroform extractions, and CsCl gradient ultracentrifugation. Since the use of chloroform should be avoided in phage preparations intended for medical use, we wanted to test whether ultrafiltration can replace PEG precipitation as the pre-purification/concentration step. To circumvent the potential health hazards associated with CsCl, we used sucrose gradient instead of CsCl in the ultracentrifugation.

**FIGURE 1:**
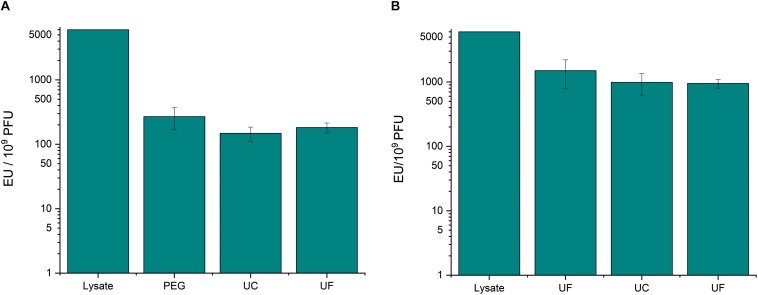
PEG precipitation and sucrose gradient ultracentrifugation in fHoEco02 purification. fHoEco02 was purified by a protocol consisting of PEG precipitation, ultracentrifugation, and ultrafiltration **(A)** or of ultrafiltration, ultracentrifugation, and ultrafiltration **(B)**. The graph shows endotoxin concentration as endotoxin unit (EU)/10^9^ PFU. Each procedure was repeated three individual times; the columns indicate the mean and error bars indicate standard deviation (SD). PEG, PEG precipitation; UF, ultrafiltration; UC, ultracentrifugation.

PEG precipitation reduced the endotoxin-to-phage ratio of fHoEco02 by ∼20 fold (Figure 1A and Supplementary Table S1), whereas ultrafiltration only by four-fold (Figure 1B and Supplementary Table S1). Thus, ultrafiltration was not as efficient method for pre-purification as PEG precipitation. However, as the phage recovery in ultrafiltration was high (Supplementary Table S1), it can be used for phage concentration and buffer exchange. Sucrose gradient ultracentrifugation only reduced the endotoxin-to-phage ratio by ∼1.5-fold (Figure 1 and Supplementary Table S1). Therefore, the endotoxin removal potential of this method was minimal.

### Removal of *E. coli* Endotoxins by Anion Exchange Chromatography and 1-Octanol Extraction

We then wanted to study how efficiently anion exchange chromatography and 1-octanol extraction remove *E. coli* endotoxins from phage preparation. To this end, two purification strategies were followed: One with ultrafiltration, anion exchange chromatography, and ultrafiltration, and another with 1-octanol extraction preceding these three steps.

**FIGURE 2:**
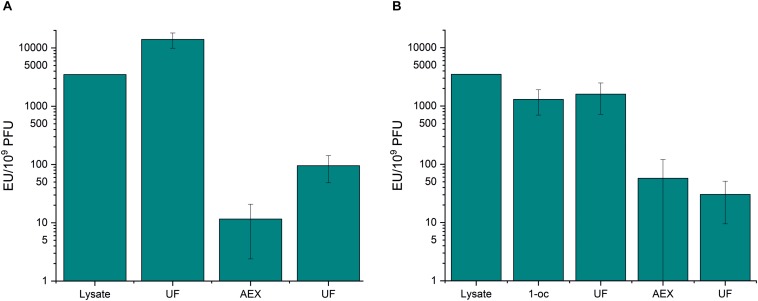
Anion exchange chromatography and 1-octanol extraction in fHoEco02 purification. fHoEco02 was purified by a protocol consisting of ultrafiltration, anion exchange chromatography, and ultrafiltration **(A)** or 1-octanol extraction, ultrafiltration, anion exchange chromatography, and ultrafiltration. **(B)** The graph shows endotoxin concentration as endotoxin unit (EU)/10^9^ PFU. Each procedure was repeated three individual times; the columns indicate the mean and error bars indicate standard deviation (SD). 1-oc, extraction with 1-octanol, UF, ultrafiltration; AEX, anion exchange chromatography.

As above, ultrafiltration did not significantly remove endotoxins from phage samples (Figure 2 and Supplementary Table S2). Anion exchange chromatography was very efficient in endotoxin removal. In the first purification strategy (ultrafiltration – chromatography - ultrafiltration), the endotoxin-to-phage ratio was lowered from 3.5 × 10^3^ EU/10^9^ PFU to 95.5 EU/10^9^ PFU (Figure 2A and Supplementary Table S2). 1-octanol extraction performed prior to the chromatography reduced the endotoxin-to-phage ratio by ∼three-fold, and the final EU/10^9^ PFU after the second purification strategy (1-octanol–ultrafiltration–chromatography–ultrafiltration), was as low as 30.4 (Figure 2B and Supplementary Table S2).

When examining the purification results and phage recoveries in individual purification steps of the first strategy (ultrafiltration–chromatography–ultrafiltration), an incoherent finding arose: In the first ultrafiltration step prior to AEX, the endotoxin-to-phage ratio seemed to rise. In addition, the phage recovery in the same step seemed very low (Supplementary Table S2). This is probably due to reversible aggregation of fHoEco02 in the low-salt chromatography buffer, leading to underestimation in the phage titer. The phenomenon of aggregation of T4 phage in low-salt conditions was recently described by Szermer-Olearnik et al. (2017). Interestingly, the same effect was not observed in the second purification strategy (1-octanol–ultrafiltration–chromatography–ultrafiltration), perhaps indicating that the residual octanol in the phage sample prevented the aggregation. In our hands, the phage recovery in octanol extraction was only 15% (Supplementary Table S2).

### Removal of *E. coli* Endotoxins by Endotoxin Affinity Columns

**FIGURE 3:**
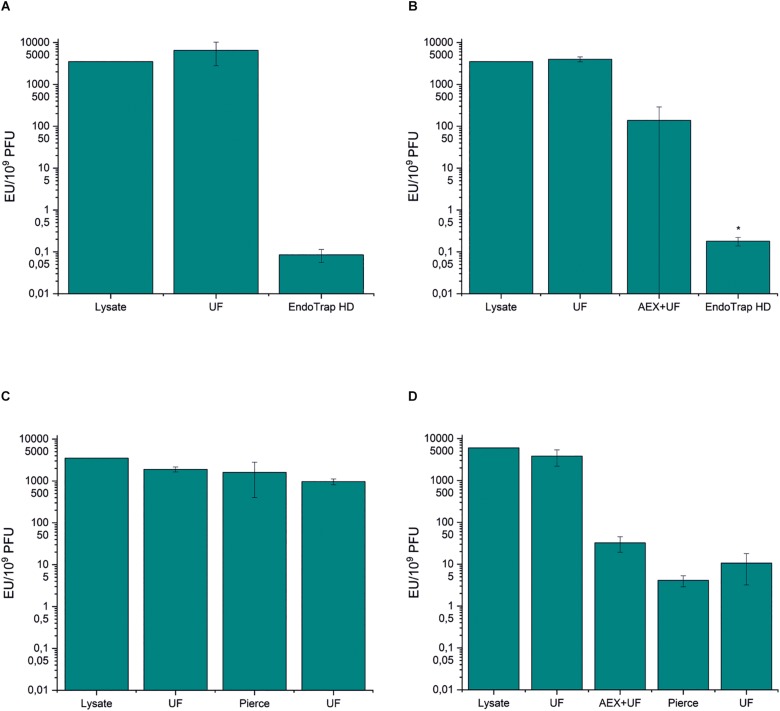
Endotoxin affinity columns and anion exchange chromatography in fHoEco02 purification. fHoEco02 was purified by a protocol consisting of ultrafiltration and EndoTrap HD column **(A)**, ultrafiltration, anion exchange chromatography, ultrafiltration, and EndoTrap HD column **(B)**, ultrafiltration, Pierce high-capacity endotoxin removal spin column and ultrafiltration **(C)**, and ultrafiltration, anion exchange chromatography, ultrafiltration, Pierce high-capacity endotoxin removal spin column, and ultrafiltration **(D)**. The graph shows endotoxin concentration as endotoxin unit (EU)/10^9^ PFU. Each procedure was repeated three individual times; the columns indicate the mean and error bars indicate standard deviation (SD). ^*^The endotoxin concentration of one out of three replicates was below the detection limit; the columns indicate the mean of two replicates and the errors indicate range. UF: ultrafiltration, AEX: anion exchange chromatography.

There are several commercial affinity columns for endotoxin removal, and in this work, we tested two of them in the purification of phage fHoEco02: EndoTrap HD and Pierce high-capacity endotoxin removal spin column. Both the columns were used either alone or in combination with ion exchange chromatography. EndoTrap HD was extremely efficient in the removal of *E. coli* endotoxins, both alone and when used after anion exchange chromatography (Figures 3A,B and Supplementary Tables S3, S4). Both strategies using EndoTrap HD resulted to endotoxin-to-phage ratio of ∼0.1 EU/10^9^ PFU, indicating that EndoTrap HD alone was enough to remove endotoxins from fHoEco02 lysate and the use of ion exchange chromatography did not add to the endotoxin removal. In contradiction, Pierce column alone did not noticeably decrease the endotoxin-to-PFU ratio (Figure 3C and Supplementary Table S3). When used after ion exchange chromatography, it reduced the ratio from 32.4 to 4.1 EU/10^9^ PFU (Figure 3D and Supplementary Table S4), indicating that it may be used for polishing of pre-purified phage samples.

Interestingly, the phage recoveries in all the steps producing extremely low endotoxin-to-phage ratios (≤4 EU/10^9^ PFU) were>100 % (Supplementary Tables S3, S4). This discrepancy may be due to receptor binding protein blocking ability of soluble LPS, causing a slight underestimation of the phage titer that is corrected when the LPS/endotoxin concentration becomes low enough. The ability of soluble LPS to inhibit phage T4 adsorption to the host bacteria has been shown earlier (Washizaki et al., 2016).

### Removal of *A. pittii* Endotoxins by Endotoxin Affinity Columns

**FIGURE 4:**
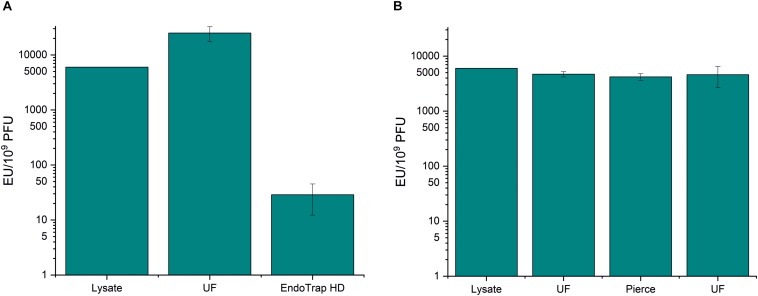
Endotoxin affinity columns and anion exchange chromatography in fHyAci03 purification. fHyAci03 was purified by a protocol consisting of ultrafiltration and EndoTrap HD column **(A)** and ultrafiltration, Pierce high-capacity endotoxin removal spin column and ultrafiltration **(B)**. The graph shows endotoxin concentration as endotoxin unit (EU)/10^9^ PFU. Each procedure was repeated three individual times; the columns indicate the mean and error bars indicate standard deviation (SD). UF, ultrafiltration.

Since most methods for endotoxin removal have been developed for *E. coli* endotoxins, we wanted to study how well the endotoxin affinity columns can purify *A. pittii* phage fHyAci03. The results shown in Figure 4 and Supplementary Table S5 indicate that the two columns tested showed similar trend with *Acinetobacter* endotoxins as with *E. coli* endotoxins: The combination of ultrafiltration and EndoTrap HD reduced the endotoxin-to-phage ratio from the 6 × 10^3^ EU/10^9^ PFU of the raw lysate down to 26.8 EU/10^9^ PFU, whereas the Pierce column had practically no effect at all. The recoveries of fHyAci03 were 143 and 58% with EndoTrap HD and Pierce columns, respectively (Supplementary Table S5), the>100% yield with EndoTrap HD perhaps reflecting the decrease of the LPS inhibition of the phage.

### Removal of Staphylococcal Enterotoxins by Ultrafiltration and Anion Exchange Chromatography

Most studies about phage purification have focused on phage yield and endotoxin removal. However, phage lysates may also contain protein toxins that can be potentially harmful to patients receiving phage therapy. To study how bacterial toxins are removed from phage products during the purification process, we analyzed the purification of *S. aureus* phage fRuSau02 cultured in a *Staphylococcus* strain that was earlier shown to produce staphylococcal enterotoxins (SEs) (Leskinen et al., 2017). The purification protocol applied here was the combination of ultrafiltration and anion exchange chromatography. To measure the SE concentration, we used an ELISA kit that measures enterotoxins A, B, C, D, and E in separate wells. The kit is not meant for quantitative analysis, but using enterotoxins A and C2 (SEA and SEC2, respectively) as standards, we found out that the kit was linear in range 0.25 to 10 ng/ml and 0.25 to 1 ng/ml for SEA and SEC2, respectively (not shown). According to the manufacturer, the detection limit of the kit was 0.25 ng/ml.

As shown in Table 1, the fRuSau02 lysate contained both SEA and SEC. Ultrafiltration reduced the ratio of both enterotoxins to phage by ∼20-fold (from 1.3 to 0.058 ng/10^9^ PFU for SEA and from 3.2 to 0.22 ng/10^9^ PFU for SEC). After anion exchange chromatography, the SE concentrations were below the detection limit of the kit.

**TABLE 1:** Ultrafiltration and anion exchange chromatography in fRuSau02 purification.

	**Lysate**	**UF**	**AEX**	**UF**
Titer (PFU/ml)	5.2 × 10^10^± 2.4 × 10^10^	2.7 × 10^10^± 8.8 × 10^9^	5.6 × 10^8^± 1.4 × 10^8^	5.0 × 10^9^± 1.6 × 10^9^
SEA (ng/ml)	53.9 ± 5.5	1.8 ± 1.3	<0.25	<0.25
SEA (ng/10^9^ PFU	1.3 ± 0.96	0.058 ± 0.035	–	–
SEC (ng/ml)	137.9 ± 12.4	5.5 ± 1.5	<0.25	<0.25
SEC (ng/10^9^ PFU)	3.2 ± 1.7	0.22 ± 0.063	–	–
Volume in (ml)	–	3.00	0.50	3.85
Volume out (ml)	–	3.00	4.00	0.48
PFU in	–	1.6 × 10^11^± 7.2 × 10^10^	1.3 × 10^10^± 4.4 × 10^9^	2.2 × 10^9^± 5.3 × 10^8^
PFU out	–	8.0 × 10^10^± 2.7 × 10^10^	2.3 × 10^9^± 5.5 × 10^8^	2.4 × 10^9^± 7.5 × 10^8^
Yield (%)	–	54 ± 11	19 ± 8.6	108 ± 7.8

*The values represent the mean and sd of three individual repetitions. Phage titers and enterotoxin concentrations indicate samples after each purification step. UF, ultrafiltration; AEX, anion exchange chromatography; –, Not possible to calculate.*

## Discussion

Due to the increasing number of infections caused by antibiotic-resistant bacteria, we face an urgent need for alternative therapies. Phage therapy is a traditional method for treating bacterial infections and it is now gaining more and more interest throughout the world as a possible solution for this need (Abedon et al., 2011; Kutter et al., 2015). To be safe for patients, phage therapy products must meet several criteria with respect to types of phages used, product purity, and the quality of their manufacture process (Pirnay et al., 2015, 2018). The aim of this work was to evaluate the usability of different phage purification methods in the production of clinical-grade phage preparations.

Phages can be administered to patients by different routes, and the purity requirements vary according to the application route used. For parenteral administration, the highest allowed endotoxin amount is 5 EU/kg of body weight/h. For a patient of 70 kg, this means 8.4 × 10^3^ EU/24 h. In a recent phage therapy case, a daily therapeutic dose of 5 × 10^9^ PFU was given intravenously to a patient having severe infection caused by *Acinetobacter baumannii* (Schooley et al., 2017). If we assume this as a typical therapeutic dose, the highest acceptable endotoxin-to-phage ratio when treating a 70-kg patient would be 1.6 × 10^3^ EU/10^9^ PFU.

The traditional method for concentration and pre-purification of phages from crude lysates is the precipitation with PEG followed by several rounds of chloroform extractions. In this study, PEG precipitation reduced the endotoxin-to-phage ratio from 6 × 10^3^ EU/10^9^ PFU of the crude filtered fHoEco02 lysate down to 269 EU/10^9^ PFU, a level sufficient for therapeutic purposes. The phage recovery in the process was 52%. However, since PEG needs to be removed from phage preparations by chloroform extraction, the method cannot be recommended for therapy purposes unless the residual chloroform concentration in the final product is analyzed and falls below the accepted level of 0.6 mg/day. As the alternative method for pre-purification, concentration, and buffer exchange, we used ultrafiltration with 100,000 MWCO cut-off. UF was fast and relatively easy to scale-up. The time consumption varied from few minutes to a couple of hours, depending on the scale and the level of the purity of the phage product. The ultrafiltration of crude lysates, especially the ones prepared from semiconfluent plates, required longer time than the buffer exchange of purified phages. Phage recoveries in ultrafiltration were usually high, even though they varied to some extent. Ultrafiltration was thus a usable method for concentration and buffer exchange, but its effect in phage purification was limited: It efficiently removed small bacterial proteins from phage products, as demonstrated here with staphylococcal enterotoxins A and C2. However, it only reduced the endotoxin-to-phage ratio minimally, resulting in values ranging from 1.5 × 10^3^ EU/10^9^ PFU to 1.4 × 10^4^ EU/10^9^ PFU (Supplementary Tables S1–S4). Therefore, ultrafiltration alone does not suffice for phages of gram-negative bacteria if parenteral administration is needed.

Perhaps to our surprise, ultracentrifugation through sucrose gradient was not very efficient in endotoxin removal. However, the combination of ultrafiltration and sucrose gradient ultracentrifugation reduced the endotoxin-to-phage ratio below the threshold of 1.6 × 10^3^ EU/10^9^ PFU, indicating that a phage product purified with this procedure might be administered parenterally. CsCl gradient is more commonly used than ultrafiltration and might be more efficient in endotoxin removal. However, as CsCl is a harmful substance (Food and Drug Administration., 2018) and its removal from phage products would increase the time consumption and laboriousness of the purification procedure, we find CsCl gradient non-optimal for medical phage production. Therefore, we did not include CsCl gradient ultracentrifugation in this study.

The combination of anion exchange chromatography and ultrafiltration resulted in phage preparations of high purity with respect to endotoxins. The endotoxin-to-phage ratios after AEX and concomitant buffer exchange by ultrafiltration were 32–140 EU/10^9^ PFU (Supplementary Tables S2, S4). The phage recoveries in AEX were generally high, however, they varied from phage to phage and even between repetitions with one phage, which was illustrated by high standard deviations after AEX purifications. A variation of phage recoveries with AEX was also reported by Adriaenssens et al. (2012). The other drawbacks of AEX are that it is rather laborious method and needs to be optimized for each phage separately. Even though a small-scale AEX run with the set-up utilized in this study (1 ml monolithic column and Äkta purifier HPLC device) takes only ∼1 h, the several washing steps required to maintain the functionality of the system increase the time consumption to ∼1 day. In addition, GMP -certified liquid chromatography devices are usually of industrial size, and therefore impractical for small-scale personalized phage therapy products. AEX used as batch purification with the ion exchange resin as a slurry might be a conceivable alternative for the production of therapeutic phage products, but the efficiency of the method should be tested in practice. To conclude, we found anion exchange chromatography to suit well for research laboratory but to be too laborious for routine therapeutical phage purification. Extraction with 1-octanol lowered the endotoxin-to-phage ratio by threefold and resulted in the endotoxin-to-phage ratio of 1.3 × 10^3^ EU/10^9^ PFU, slightly lower than the threshold 1.6 × 10^3^ EU/10^9^ PFU required for parenteral administration. The drawbacks of 1-octanol were the low phage recovery rate (15 % with fHoEco02, Supplementary Table S2) and the need to remove the solvent, which slows down the process (Bonilla et al., 2016). In our hands, the phage recoveries with octanol extractions were slightly lower than those in earlier studies (Szermer-Olearnik and Boratynski, 2015; Bonilla et al., 2016), which may indicate that different phages tolerate the solvent differently.

In this study, EndoTrap HD column was the optimal way to remove endotoxins from phage preparations. The use of the column needed only little hands-on-time and resulted in extremely low endotoxin-to-phage ratio with high phage recovery. In addition, the scale-up is straightforward. The purification of *E. coli* phage produced lower endotoxin concentration than that of the *A. pittii* phage, but even the fHyAci03 product was pure enough for parenteral administration. To our surprise, the Pierce endotoxin removal column behaved very differently: Even though the phage recoveries were good, there was only minimal improvement in the endotoxin-to-phage ratio. The binding capacities of the two columns, as stated by the manufacturers, were 5,000,000 and 2,000,000 EU/ml for EndoTrap HD and Pierce columns, respectively. The total endotoxin amounts that we applied to the columns were ∼10^4^ EU/ml, which is clearly below the reported limits. Therefore, the poor result with the Pierce column was not explained by exceeding the binding capacity. Interestingly, the outcome with the Pierce column was similar to the finding by Cooper et al., 2014, who failed to remove endotoxins from a phage cocktail targeting *P. aeruginosa* using EndoTrap Blue column (Cooper et al., 2014). Without knowing the exact molecules used as the affinity ligands in these columns, it is not possible to elucidate the precise reason for their different performance. However, we hypothesize that the affinity of the Pierce and EndoTrap Blue columns to endotoxins is too weak to detach LPS molecules that are attached to the tail proteins of the phages, whereas the affinity of EndoTrap HD to endotoxins seems to be high enough.

There are no strict limits for the concentrations of staphylococcal enterotoxins in medical products. However, Asao et al. (2003) estimated that 20–100 ng of enterotoxin A was enough to cause food poisoning (Asao et al., 2003). In this study, the enterotoxin-to-phage ratio in unpurified fRuSau02 lysate was 1.3 ng/10^9^ PFU for SEA and 3.2 ng/10^9^ PFU for SEC. The therapeutic dose of 5 × 10^9^ PFU would thus contain 6.5 ng of SEA and 16 ng of SEC, which might cause symptoms to the most sensitive patients when administered orally. Ultrafiltration reduced the enterotoxin-to-phage ratio by ∼20-fold, resulting to a phage preparation that would be safe for oral administration with respect to enterotoxins.

## Data Availability

All datasets generated for this study are included in the manuscript and/or the Supplementary Files.

## Author Contributions

SK designed the work. VH, JH-H, and AC performed the experiments. VH, JH-H, AC, and SK analyzed the data. VH, MS, and SK wrote the manuscript.

## Conflict of Interest Statement

The authors declare that the research was conducted in the absence of any commercial or financial relationships that could be construed as a potential conflict of interest.
